# *Echinococcus granulosus* complex infection in wild boar hunters and auxiliary hunting dogs

**DOI:** 10.1016/j.onehlt.2024.100908

**Published:** 2024-10-04

**Authors:** Mariaelisa Carbonara, Francesco Buono, Anna Morea, Giovanni Sgroi, Maria Paola Maurelli, Francesco Locantore, Paolo Trerotoli, Francesca Indraccolo, Angela Stufano, Valentina Schino, Nicola D'Alessio, Vincenzo Veneziano, Piero Lovreglio, Domenico Otranto, Roberta Iatta

**Affiliations:** a Department of Veterinary Medicine, University of Bari Aldo Moro, Italy; b Department of Veterinary Medicine and Animal Production, University of Naples Federico II, Naples, Italy; c Interdisciplinary Department of Medicine, University of Bari Aldo Moro, Bari, Italy; d Department of Animal Health, Istituto Zooprofilattico Sperimentale del Mezzogiorno, Portici, Italy; e Department of Preventive Medicine, ASL Foggia SIAV A Nord, Italy; f Department of Veterinary Clinical Sciences, City University of Hong Kong, SAR, China

**Keywords:** Cystic echinococcosis, *Echinococcus granulosus* sensu lato, Wild boar hunting, Hunting dogs, Infection risk

## Abstract

Cystic echinococcosis (CE) caused by *Echinococcus granulosus* sensu lato (s.l.) is a zoonotic neglected tropical disease endemic in Italy, which perpetuates in several intermediate hosts, including wild boars, and dogs as definitive hosts. People living in rural and livestock-raising areas are exposed to *E. granulosus* s.l. infection, as well as people leading outdoor activities in endemic regions. Therefore, this study was designed to assess the exposure to *Echinococcus* spp. in wild boar hunters, the role of their hunting dogs as parasite reservoirs, along with hunter's knowledge on the infection risk.

From December 2022 to May 2023, wild boar hunters (*n* = 122) from southern Italy were recruited on volunteer basis for blood and serum sampling and a questionnaire enquiring socio-demographic, anamnestic data and knowledge on CE was also filled out. Sera were tested for *Echinococcus* spp. IgG by a commercial enzyme-linked immunosorbent assay (Euroimmun ELISA®, Germany). In addition, faecal samples from their hunting dogs (*n* = 208) were screened for Taeniidae eggs by parasitological and molecular approaches.

Overall, six (4.9 %) hunters scored either positive or borderline for IgG anti-*Echinococcus* spp., of which one presented a calcified hepatic cyst at abdominal ultrasonography. In addition, 6.3 % Taeniidae prevalence was recorded in faecal samples (13/208) of hunting dogs, and *E. granulosus* sensu stricto (s.s.) was molecularly identified in two samples. The statistical analysis revealed the risk factors (odds ratio > 1, *p* < 0.05) associated with parasitic exposure, including the hunter geographical provenience, and the presence of animals around or in the house.

The *E. granulosus* s.l. exposure of hunters herein detected, coupled with the parasite molecular positivity of their hunting dogs and the limited awareness on *Echinococcus* spp. life cycle/infection risk, highlight the relevance to promote health surveillance and educational programs within the hunting category, for minimizing the cestode circulation in the wildlife-urban premises.

## Highlights

• Cystic echinococcosis may occur in wildlife/*peri* urban settings.
• Overall, 4.9 % wild boar hunters were exposed to *Echinococcus* spp.
• *Echinococcus granulosus* DNA was detected in two hunting dog faecal samples.
• Hunters had fragmented awareness on *E. granulosus* life cycle/infection risk.
• Health surveillance and educational programs for wild boar hunters are advocated.

## Graphical abstract

**Figure:**
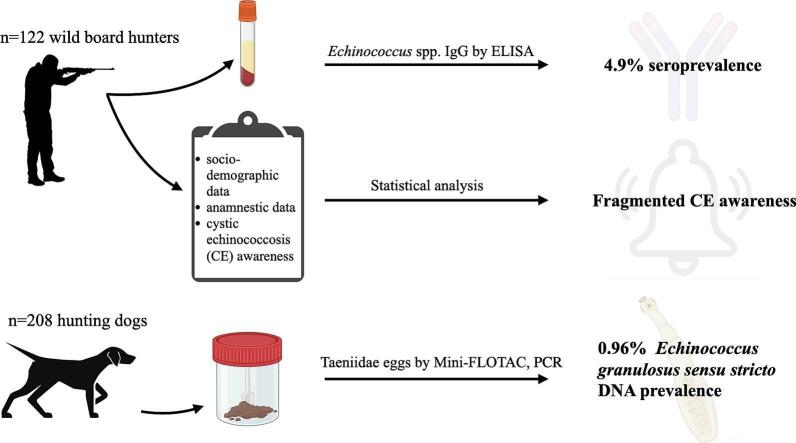

## 1 Introduction

Cystic echinococcosis (CE), also known as hydatidosis, is a zoonotic neglected tropical disease caused by the tapeworm *Echinococcus granulosus* sensu lato (s.l*.*) [1]. With a worldwide geographical distribution, CE occurs in rural and pastoral communities (e.g., livestock-raising areas) in both low- and upper-middle-income countries, mainly in China and central Asia, South America, Africa, Australia, eastern and southern Europe [2].

The tapeworm is naturally transmitted through oral-faecal and predator-prey routes, between canids (definitive hosts) and ungulates, especially sheep, which act as intermediate hosts [3,4]. Humans are dead-end hosts for *E. granulosus* s.l., becoming infected after the accidental ingestion of raw vegetables or water contaminated by eggs. After infection, larval stages develop in internal organs, mainly liver and lungs, as thick-walled and fluid-filled cysts [5]. The hydatid cysts, as long as they measure less than 10 cm and occupy less than 70 % of the organ volume, may not induce human tissue damage, leading to a chronic and asymptomatic infection, which can last even 15 years [1]. Given all the above, the CE diagnosis in humans represents a complex task. Accordingly, the detection of *E. granulosus* s.l. cysts in patients is usually an accidental finding, following imaging investigations (i.e., ultrasound, X-ray) performed for other pathologies and further confirmed by serological tests, such as enzyme-linked immunosorbent assay (ELISA) [6,7]. As a result, the real burden of CE is largely underestimated by the national health systems, although it is a notifiable infectious disease in most of the European countries [8]. Specifically, in 2012 the Italian Register of CE (RIEC) was launched and after two years expanded into the European Register of CE (ERCE), aiming to improve the epidemiology, diagnosis and control of the disease [8]. To date, Italy is classified as a high endemic area for CE, as defined by World Health Organization, with a mean annual incidence of 1.21 per 100.000 inhabitants in the period 1997–2020 [2].

The asymptomatic presentation of *E. granulosus* s.l. infection in dogs, coupled with the low specificity and sensitivity of common coprological examinations in detecting the tapeworm eggs, contribute to the underestimation of the parasite circulation [9,10]. Accurate methods for the diagnosis of canine intestinal *E. granulosus* infections are based on serological or molecular tests, which are also employed for research purposes [9,11].

As far the CE risk, people living in rural and livestock-raising areas, as well as those leading outdoor activities in areas contaminated by parasitic eggs, may be exposed to *E. granulosus* s.l. infection. Wild boar hunting, an ancient habit extremely common in Italy [12], poses a risk of the parasitic infection for hunters, as they are potentially exposed to eggs shed by their dogs [13]. Noteworthy, a CE prevalence of 4.4 % was recorded in wild boars from southern Italy highlighting their role in spreading this cestode into the wild settings [14]. Meanwhile, an overall *Echinococcus* seroprevalence of 11 % was detected in hunters from Poland [15]. However, to the authors' knowledge, studies investigating the *E. granulosus* s.l. infection prevalence in both hunters and their auxiliary hunting dogs, in CE endemic areas, are still lacking.

Given the above, this study aimed to assess the seroprevalence of *Echinococcus* spp. in wild boar hunters in southern Italy, the role of their hunting dogs as parasite reservoirs, as well as the hunter's knowledge on the CE and the infection risk.

## 2 Materials and methods

### 2.1 Hunter sampling and serological testing

From December 2022 to May 2023, 122 wild boar hunters from Basilicata (*n* = 90) and Campania (*n* = 32) regions (southern Italy) were recruited on volunteer basis by attending health care facilities and filling out a questionnaire. This form was divided in two sections which included: i) socio-demographic, anamnestic data and information about their hunting dogs, and ii) general knowledge on *E. granulosus* s.l. infection. After completing the questionnaire, each hunter underwent blood and serum sampling collected in individual vials and then stored at −20 °C until laboratory analysis. Each serum was tested in double for *Echinococcus* spp. IgG by a commercial enzyme-linked immunosorbent assay (Euroimmun ELISA®, Germany). The microplate was read at the absorbance of 450 nm and the results expressed as ratio of the absorbance of the control and the calibrator. Samples with ratio higher than 1.1 were considered as positive, between 0.8 and 1.1 as borderline, and less than 0.8 as negative. Seropositive/borderline patients were asked to undergo imaging investigation by abdominal ultrasonography (US) for checking the presence of hydatid cysts.

### 2.2 Sampling of hunting dogs and coprological examinations

From February to June 2023, faecal samples were collected from 208 hunting dogs from Basilicata (*n* = 170) and Campania regions (*n* = 38) owned by 100 hunters enrolled (see above). The hunters were instructed to keep their dogs on leash and/or in single box for at least 1 day prior to sampling. The stools were stored in portable refrigerators (at 4 °C) and copromicroscopic examination performed within 48 h after collection. All faecal samples were firstly macroscopically observed for detecting tapeworm proglottids and then, two grams of each sample analyzed by a quali-quantitative coprological technique (Mini-FLOTAC) with a detection limit of five eggs/cyst/oocyst/larvae per gram of feces (EPG/CPG/OPG/LPG) [16]. The floatation medium used was a zinc sulphate (ZnSO_4_) solution with a specific gravity of 1.350 [17]. Eggs were morphologically identified using taxonomic keys [18,19] and individual faecal egg counts (FECs) performed.

### 2.3 Molecular detection of cestodes in faecal samples

DNA extraction was performed from each faecal sample using the QIAamp Stool Mini Kit (Qiagen, Germany), according to the manufacturer's instructions. A multiplex PCR protocol was used to detect and identify *E. granulsosus* and *Taenia* spp. positive samples, amplifying 117 bp and 267 bp fragments of the small subunit of ribosomal RNA (rrnS) respectively, using the PCR thermal profile previously described [20]. A 50 μl of PCR reaction mix was prepared, containing 1× EmeraldAmp MAX PCR Master Mix (Takara, Japan), 2 μM of primers Cest1, Cest2, Cest3, Cest4, 16 μM of primer Cest5 and 2 μl of template DNA. Moreover, to confirm results obtained by the rrnS PCR, a second end point PCR was used to amplify 529 bp of the mitochondrial cytochrome *c* oxidase subunit I (*cox*1) gene of *E. granulosus* positive samples, following the thermal profile formerly described [21]. A 50 μl of PCR reaction mix was prepared, containing 1× EmeraldAmp MAX PCR Master Mix (Takara, Japan), 25 pmol of primers JB3 and JB4.5 and 5 μl of template DNA.

For both conventional PCRs (cPCRs), amplified products were visualized on a 2 % ethidium bromide-stained low melting agarose gel (BIO-RAD, Spain). DNA bands were cut from the gel, purified by QIAquick Gel Extraction KIT (Qiagen, Germany) and sequenced in both forward and reverse directions. Sequencing analysis was performed, using the Chromas version 2.6.6 software and compared with sequences in the GenBank database, using BLAST system.

### 2.4 Statistical analysis

Exact binomial 95 % confidence intervals (CIs) were established for each prevalence recorded. Fisher test was applied to assess statistical differences of prevalence of seropositive hunters and variables (i.e., age, dwelling type, living setting, presence of animals in/around the house, number of dogs owned, contact among hunting dogs and other animals) with statistical significance at *p*-value (p) less than 0.05. Odds ratios (ORs) were calculated to assess the infection risk according to the variables of the participants and the questionnaire answers. A multivariable exact conditional logistic regression was applied to determine adjusted ORs. The dependent variable was the hunter seropositivity, and the independent variables were all the characteristics observed in the studied population. The selection of the variables was performed by a forward selection. The criteria to entry the model was a *p*-value = 0.05 for the Score Statistic of the full model. The 95 % CIs, chi-square, *p*-value and OR values were calculated by using the software Epitools - Epidemiological Calculators [22], MEdCalc [23], and SAS 9.4 for PC LOGISTIC procedure (SAS Institute Inc., Cary, NC, USA).

## 3 Results

Out of 122 hunters enrolled, the majority were older than 50 years and 37 % of them owned more than five hunting dogs each (Table 1). Overall, six (i.e., 4.9 %, 95 % CI: 2.3–10.3 %) hunters from Basilicata scored either positive (*n* = 3) or borderline (n = 3) for IgG anti-*Echinococcus* spp., and two of them (i.e., one positive and one borderline) underwent imaging investigation. The seropositive hunter showed a calcified 4 mm diameter hepatic cyst at abdominal ultrasonography. No hunters tested seropositive in Campania region. All the details regarding the characteristics of seropositive/borderline hunters are reported in Table 2.

**Table 1:** Univariate and multivariate analyses of risk factors and their association with *Echinococcus granulosus* s.l*.* seropositivity in wild boar hunters.

Variable	Category	N. positive/tested (%)	Univariate analysis		Multivariate analysis	
			OR (95 % CI)	*p*-value	OR (95 % CI)	*p*-value
Age (years)	≤50	3/50 (6.0)	1.44 (0.3–7.4)	0.69	–	
	>50	3/72 (4.2)				
Geographical provenience (region)	Basilicata	6/90 (6.7)	5.18 (0.3–94.6)	0.2669	72.6 (11.1-inf.)	<0.0001
	Campania	0/32 (0.0)				
Dwelling type	country house	3/44 (6.8)	1.77 (0.3–9.2)	0.67	–	
	apartment	3/78 (3.8)				
Living setting	rural	3/44 (6.8)	1.77 (0.3–9.2)	0.67	–	
	urban	3/78 (3.8)				
Presence of animals in/around the house	only others (e.g. horses, pigs, ruminants)	3/7 (7.1)	38.25 (3.2–457.4)	0.004	17.1 (2.1-inf.)	0.0114
	only dogs	1/25 (2.2)	2.12 (0.13–35.44)	0.5996	–	
	only cats	0/16 (0.0)	1.04 (0.04–26.78)	0.9809	–	
	at least 2 categories	1/18 (5.6)	3 (0.18–50.62)	0.446		
	3 categories	0/4 (0.0)	3.81 (0.13–107.9)	0.4324		
	none	1/52 (1.9)	*-reference-*			
Contact hunting dogs-other animals	other dogs	1/6 (16.7)	14.6 (0.79–269.7)	0.0716	–	
	others (ruminants, pigs, horses)	3/42 (7.1)	7.68 (0.82–71.18)	0.0726	–	
	none	2/52 (3.8)	*-reference-*			
Canine preventive antiparasitic treatments	vs ectoparasites	0/10 (0.0)	2.9 (0.05–155.8)	0.5997	–	
	vs gastrointestinal helminths	0/8 (0.0)	3.6 (0.07–194.6)	0.5306	–	
	combined	6/74 (8.1)	5.8 (0.3–106.03)	0.2366	–	
	none	0/8 (0.0)	*-reference-*			
Awareness about eating raw vegetables and/or direct contact with dogs	no	4/83 (4.8)	1.07 (0.18–6.09)	0.9413	–	
	yes	2/39 (5.1)				
Awareness about transmission route of *E. granulosus* s.l. to dogs	yes	5/42 (11.9)	10.7 (1.2–94.6)	0.0334	–	
	no	1/80 (1.2)				
Proper disposal of wild boar's viscera	yes	1/13 (7.7)	1.7 (0.18–16.09)	0.6286	–	
	no	5/109 (4.5)				
Chest pain	yes	0/8 (0.0)	0.98 (0.05–18.95)	0.9904	–	
	no	6/114 (5.3)				
Abdominal pain	yes	0/10 (0.0)	0.78 (0.04–14.84	0.8688	–	
	no	6/112 (5.4)				
Spleen/liver disorders	yes	0/7 (0.0)	1.12 (0.06–21.89)	0.9389	–	
	no	6/115 (5.2)				
Immune system pathologies	yes	0/5 (0.0)	1.56 (0.08–31.36)	0.7717	–	
	no	6/117 (5.1)				
Heart valve pathologies	yes	0/5 (0.0)	1.56 (0.08–31.36)	0.7717	–	
	no	6/117 (5.1)				
Cardiac arrhythmias	yes	0/6 (0.0)	1.31 (0.07–25.83)	0.8601	–	
	no	6/116 (5.2)				
Lymphadenopathy	yes	0/1 (0.0)	5.92 (0.22–160.07)	0.2902	–	
	no	6/121 (5.0)				
Alcohol user	yes	6/92 (6.5)	4.58 (0.25–83.81)	0.3045	–	
	no	0/30 (0.0)				
Smoking	yes	2/43 (4.6)	0.91 (0.16–5.21)	0.9199	–	
	no	4/79 (5.1)				

Note: “-“not entered in the model because didn't meet the entry criteria in the multivariable forward selection (Score chi-square = 30; p < 0.0001).

“reference”: the comparator for the odds ratio determination in those categorical variables with more than two classes.

**Table 2:** Diagnostic and anamnestic data of *E. granulosus* s.l. seropositive hunters, according to their awareness about: A) transmission route of *E. granulosus* s.l. to humans, B) transmission route of *E. granulosus* s.l*.* to dogs, C) harvest of hunting dog's feces, D) proper disposal of wild boar's viscera.

Hunter ID	Serological result	Age (years)	Animal related job	N. owned dogs	A	B	C	D	Imaging investigation	Alterations
1	borderline	64	no	0	no	no	no	no	no	
2	seropositive	61	yes	3	no	yes	no	yes	no	
3	borderline	39	no	8	no	yes	no	no	no	
4	borderline	24	no	7	no	yes	no	no	yes	none
5	seropositive	66	no	2	yes	yes	no	no	no	
6	seropositive	22	no	3	no	yes	no	no	yes	hepatic calcification

At the microscopic and molecular examinations of canine faecal samples (Table 3), the overall prevalence for Taeniidae was 6.3 % (i.e., 13/208; 95 % CI: 3.7–10.4), of which 5.3 % (11/208, 95 % CI: 3.0–9.2) scored positive for *Taenia* spp. and 0.96 % (2/208, 95 % CI: 0.26–3.4) for *E. granulosus* sensu stricto (s.s.). Twelve (i.e., 7.1 %, 12/170, 95 % CI: 4.1–11.9) hunting dogs from Basilicata scored positive for Taeniidae eggs, with a mean EPG count of 34.6 ± 54.7 (min. 5 – max. 175), of which eleven were positive for *Taenia* spp. and the remaining one for *E. granulosus* by rrnS multiplex PCR. Furthermore, one dog from Campania (i.e., 2.6 %, 1/38, 95 % CI: 0.5–13.5), negative at coprological examination, scored molecularly positive for *E. granulosus*. No proglottids were macroscopically observed in any canine faecal sample. All the remaining samples scored molecularly negative for Taeniidae DNA. *Echinococcus granulosus* DNA positive samples were further confirmed by cPCR targeting *cox*1 gene and sequenced. At Blast analysis, two *cox*1 gene sequences showed 100 % nucleotide identity with *E. granulosus* s.s*.* genotype 3 (MK780854), and eleven rrnS gene sequences with *Taenia hydatigena* (AB031352). The sequences obtained were submitted to GenBank database with accession no PQ178169 and PQ179694 for *E. granulosus* s.s. from Campania and Basilicata, respectively and PQ186849 for *T. hydatigena.* No *Echinococcus* spp. seropositive hunter owned a dog positive to *T. hydatigena* or *E. granulosus* s.s.

**Table 3:** Coprological and molecular data of canine faecal samples positive for Taeniidae.

Dog ID	Origin (region)	Macroscopical observations	Microscopical observations	Egg per gram count	rrnS multiplex cPCR results	rrnS sequences, % nucleotide identity with accession number	*cox*1 sequences, % nucleotide identity with accession number
1	Basilicata	none	Taeniidae eggs	175	*Taenia* spp.	*Taenia hydatigena*, 100 % AB031352	
2	Basilicata	none	Taeniidae eggs	10	*Taenia* spp.	*Taenia hydatigena*, 100 % AB031352	
3	Basilicata	none	Taeniidae eggs	5	*Taenia* spp.	*Taenia hydatigena*, 100 % AB031352	
4	Basilicata	none	Taeniidae eggs	120	*Taenia* spp.	*Taenia hydatigena*, 100 % AB031352	
5	Basilicata	none	Taeniidae eggs	5	*Taenia* spp.	*Taenia hydatigena*, 100 % AB031352	
6	Basilicata	none	Taeniidae eggs	20	*Taenia* spp.	*Taenia hydatigena*, 100 % AB031352	
7	Basilicata	none	Taeniidae eggs	25	*E. granulosus*	–	*E. granulosus* s.s.*,*100 % MK780854
8	Basilicata	none	Taeniidae eggs	5	*Taenia* spp.	*Taenia hydatigena*, 100 % AB031352	
9	Basilicata	none	Taeniidae eggs	10	*Taenia* spp.	*Taenia hydatigena*, 100 % AB031352	
10	Basilicata	none	Taeniidae eggs	30	*Taenia* spp.	*Taenia hydatigena*, 100 % AB031352	
11	Basilicata	none	Taeniidae eggs	5	*Taenia* spp.	*Taenia hydatigena*, 100 % AB031352	
12	Basilicata	none	Taeniidae eggs	5	*Taenia* spp.	*Taenia hydatigena*, 100 % AB031352	
13	Campania	none	none		*E. granulosus*	–	*E. granulosus* s.s*.,*100 % MK780854

By the questionnaire analysis, Table 1 none of the seropositive hunters referred chest/abdominal pain or spleen/liver disorders (Table 1). At the univariate analysis, the ORs referred to hunters younger than 50 years old (OR = 1.44, *p* = 0.69), living in Basilicata (OR = 5.18, *p* = 0.2669), in country house (OR = 1.77, *p* = 0.67) and in rural areas (OR = 1.77, p = 0.67), owners of hunting dogs in contact with other dogs (OR = 14.6, *p* = 0.0716) and animals (OR = 7.68, *p* = 0.0726) and owning more than 5 hunting dogs (OR = 1.31) showed higher chances of being infected, although not statistically significant. Whereas the presence of other animals (such as horses, pigs, ruminants) around the house resulted as a risk factor in the univariate analysis (OR = 38.25, *p* = 0.004) (Table 1). Most of the seropositive hunters were aware about parasitic transmission route to humans (OR = 1.07) and to dogs (OR = 10.7) (Table 1, Table 2). The risk factors confirmed by the multivariate analysis as statistically significant were living in Basilicata region (OR = 72.6, *p* < 0.0001) and the presence of animals in or around the house (OR = 17.1, *p* = 0.0114).

In addition, by assessing the CE knowledge of the enrolled hunter population, only the 33.6 % and 34.4 % of the hunters interviewed knew the transmission route of *E. granulosus* s.l. to humans and dogs, respectively, and 24.6 % and 10.6 % the correct practice of the disposal of hunting dog's feces and wild boar's viscera, respectively (Table 4).

**Table 4:** Hunter knowledge on cystic echinococcosis.

Question	Answer	N hunters (%)
How *E. granulosus* s.l. is transmitted to humans?	I don't know	61 (50.0)
	Consumption of raw meat	19 (15.6)
	Consumption of raw viscera	1 (0.8)
	Consumption of raw vegetables and/or direct contact with dogs	41 (33.6)
How *E. granulosus* s.l*.* is transmitted to dogs?	I don't know	57 (46.7)
	Consumption of raw meat	18 (14.8)
	Consumption of raw viscera	42 (34.4)
	Other (e.g. tick, animal bite)	5 (4.1)
Did you observe any cysts in wild board viscera?	Yes	43 (35.2)
	No	79 (64.8)
If yes, which were their disposal?	Feeding dogs	5 (11.5)
	Left in the field	22 (51.2)
	Delivery to veterinary services	13 (30.0)
	Other (e.g., burned, buried)	3 (7)
Where dogs live?	In the house	3 (3)
	Outside the house	91 (91)
	In both	6 (6)
Where dogs defecate?	In the countryside close to the house	97 (97)
	In the backyard of the house	3 (3)
Which is the frequency of feces disposal?	Never	37 (37)
	Sometimes	29 (29)
	Often	9 (9)
	Always	25 (25)

## 4 Discussion

This study represents the first investigation on *E. granulosus* s.l. infection in both exposed hunters and auxiliary hunting dogs, providing an epidemiological overview on CE in southern Italy. Data presented suggest that wild boar hunters are scarcely aware of the parasite life cycle and of the infection risk, spotting the need of health educational programs for minimizing the *E. granulosus* s.l. infection.

The overall *Echinococcus* spp. seroprevalence recorded in wild boar hunters from Basilicata (i.e., 4.9 %) is higher than that retrieved among polish foresters (i.e., 3.2 %) [24], or randomly selected individuals from Spain (i.e., 3.4 %) [25] and Greece (i.e., 1.1 %) [26], suggesting a high risk of exposure of the hunting category, which is likely related to their close contact with both dogs and wildlife. Accordingly, the 4.4 % CE prevalence in wild boars hunted in southern Italy, as well as the common practice among hunters to feed dogs with raw offal [14,27,28] contribute to the perpetuation of the parasitic semi-domestic life cycle. In addition, for economic reasons, wild boar hunters usually treat their dog packs with injectable or oral macrocyclic lactones (i.e., mainly ivermectin) registered for cattle, thus favoring the perpetuation of the biological cycle of cestodes [29]. Conversely, the seronegativity in hunters from Campania region might be related to the higher awareness of veterinarians about CE monitoring in wild boars as well as to the many educational initiatives which may have raised the awareness of hunters on *Echinococcus* spp. life cycle and infection risks [14].

Nonetheless, the overall human seroprevalence herein recorded might be underestimated, given the low sensitivity of serological assays in detecting *Echinococcus* spp. human exposure [30,31]. Indeed, the embedding of the metacestode may not trigger the humoral immune response, resulting in absence of detectable antibodies [31]. Therefore, patients scoring doubt at serodiagnosis as well as people at high risk of infection, should undergo imaging investigation, including US examination, which is considered the choice method for early CE diagnosis, although challenging in detecting small cysts [1,2].

On the other hand, the coprological prevalence of *E. granulosus* s.s. recorded in hunting dogs is difficult to be compared with literature data being influenced by diagnostic test employed (e.g., coproantigen ELISA, necropsy, egg isolation and molecular tests), type of dog population analyzed (e.g., farm, shepherd, free-ranging dogs) and CE endemicity level in the areas investigated [3,32]. As a matter of fact, the low prevalence herein detected in dogs by coprology (i.e., 0.96 %, 2/208) might be due to the lower sensitivity of this method when compared with coproantigen ELISA (i.e., 8 % in Spain; up to 31 % in Italy) [9,33] or with direct parasite detection in digestive tract of dogs (i.e., 2.7 % in Albania) [34]. The finding of *E. granulosus* s.s. G3 in hunting dogs feces is in accordance with literature data reporting the sympatric occurrence of the main genotype G7 with G1 and G3 in wild boars from central-southern Italy [[35], [36], [37]].

Although farm and shepherd dogs are more exposed to *Echinococcus* spp. infection than hunting ones [9,38], both canine groups live in the countryside and their physical activity is often not supported by a proper nutritional intake and shelter, which may favor the parasitic infection and spreading [39]. Considering the above, as well as the fact that countryside working dogs are not regularly health checked and treated with preventive products (e.g., vaccinations, antiparasitic treatments), educational programs on the appropriate management of these animals are recommended in countryside areas [1]. Noteworthy, the seropositivity in hunters owned dogs negative for *E. granulosus* s.s. might depend on the short time of shedding by dogs [40] as well as by the fact that humans often become infected by ingesting contaminated food or water [1].

The finding that none of the seropositive hunters referred any clinical disorder is consistent with the chronic and asymptomatic course of human CE [1], as diagnosed clinical cases represent a small proportion of the total burden of infected individuals [41]. Hence, the asymptomatic clinical presentation of humans infected by *E. granulosus* coupled with the delayed and/or missed CE diagnosis [2], make challenging to link the occurrence of human hydatidosis with the exposure to wildlife, further supporting the importance to perform regular health care checks within the exposed category [15].

The higher seroprevalence in hunters younger than 50 years old (OR = 1.44), although not statistically significant, suggests that the exposure to *Echinococcus* spp. may occur in young individuals, eventually followed by clinical manifestation or the accidental detection of hydatid cysts during routine imaging investigations [1,42,43]. In this specific context, the possibility that elder hunters, being involved in more hunting seasons, may have a higher risk of exposure to the cestode cannot be ruled out.

The *Echinococcus* infection risks (i.e., OR > 1) associated with hunters living in rural areas/country houses, as well as to those owning more than five hunting dogs each, live in presence of different animal species, including dogs, further support the association between CE epidemiology and socio-demographic variables that may favor the parasite transmission, especially if scant hygiene practices occur [44].

Although, most of the seropositive hunters knew the route of parasite transmission to dogs and humans, the low CE awareness of the whole hunter population interviewed, the great unconsciousness on the *E. granulosus* s.l. life cycle and on the correct managing of canine stools/wild boar's organs, represent an important gap towards the control of the infection [14]. In particular, the abandonment of wild boar viscera in the field is pivotal for maintaining the enzooticity of human infection, mainly when hunting and roaming dogs occur.

Considering the prevalence of *E. granulosus* reported in the wolf population from northern Italy (5.6 %) [45] and the increasing in wolf populations in southern Italy [46], future studies should assess their potential role in maintaining the cestode life cycle.

Finally, considering the study limitations, such as the enrolling population performed on volunteer basis, the use of serology as the only screening test and the low number of seropositive hunters recorded, studies on human CE in professional categories and population at risk should be implemented by using more sensible diagnostic tools (i.e., portable ultrasonography) to achieve an accurate epidemiological picture.

## 5 Conclusions

This study provides an overview on the CE epidemiology in southern Italy, highlighting the *E. granulosus* s.l*.* exposure of wild boar hunters and the role of their dogs as parasite reservoirs. In addition, the fragmented CE awareness of hunters advocates for public health education campaigns on *Echinococcus* life cycle and infection risk as well as for routine abdominal ultrasonography-based surveillance and strategic hunting dog deworming.

## Ethical statement

The study was approved by the ethics committee of the University Hospital of Bari (Italy) (approval no. 7770, protocol no. 0053676–08062023).

## Funding

This research did not receive any specific grant from funding agencies in the public, commercial, or not-for-profit sectors.

## CRediT authorship contribution statement

**Mariaelisa Carbonara:** Writing – review & editing, Writing – original draft, Methodology, Formal analysis, Data curation, Conceptualization. **Anna Morea:** Writing – review & editing, Methodology. **Giovanni Sgroi:** Writing – review & editing, Methodology. **Maria Paola Maurelli:** Writing – review & editing, Methodology, Data curation, Conceptualization. **Paolo Trerotoli:** Formal analysis, Writing – review & editing. **Francesca Indraccolo:** Writing – review & editing, Methodology. **Angela Stufano:** Writing – review & editing, Methodology, Data curation. **Valentina Schino:** Writing – review & editing, Methodology. **Nicola D'Alessio:** Writing – review & editing, Methodology, Data curation. **Vincenzo Veneziano:** Writing – review & editing, Methodology, Investigation, Formal analysis, Data curation. **Piero Lovreglio:** Writing – review & editing, Methodology, Data curation. **Domenico Otranto:** Writing – review & editing, Writing – original draft, Methodology, Data curation. **Roberta Iatta:** Writing – review & editing, Writing – original draft, Supervision, Methodology, Investigation, Formal analysis, Data curation, Conceptualization.

## Declaration of competing interest

The authors declare that they have no known competing financial interests or personal relationships that could have appeared to influence the work reported in this paper.

## Data Availability

All the sequences generated in this study will be deposited in the GenBank database. Raw data are available from the corresponding author, upon reasonable request.
